# Electrostatic Sensor Application for On-Line Monitoring of Wind Turbine Gearboxes

**DOI:** 10.3390/s18103574

**Published:** 2018-10-22

**Authors:** Huijie Mao, Hongfu Zuo, Han Wang

**Affiliations:** College of Civil Aviation, Nanjing University of Aeronautics and Astronautics, Nanjing 210016, China; rmss@nuaa.edu.cn (H.Z.); wh_rms@126.com (H.W.)

**Keywords:** electrostatic sensor, wind turbine, oil debris monitoring, gearbox, lubrication system

## Abstract

The oil-line electrostatic sensor (OLES) is a new online monitoring technology for wear debris based on the principle of electrostatic induction that has achieved good measurement results under laboratory conditions. However, for practical applications, the utility of the sensor is still unclear. The aim of this work was to investigate in detail the application potential of the electrostatic sensor for wind turbine gearboxes. Firstly, a wear debris recognition method based on the electrostatic sensor with two-probes is proposed. Further, with the wind turbine gearbox bench test, the performance of the electrostatic sensor and the effectiveness of the debris recognition method are comprehensively evaluated. The test demonstrates that the electrostatic sensor is capable of monitoring the debris and indicating the abnormality of the gearbox effectively using the proposed method. Moreover, the test also reveals that the background signal of the electrostatic sensor is related to the oil temperature and oil flow rate, but has no relationship to the working conditions of the gearbox. This research brings the electrostatic sensor closer to practical applications.

## 1. Introduction

As an important subsystem of wind turbines, gearbox maintenance costs are considered to be a leading component of the operation and maintenance (OM) costs of a wind turbine, involving cranes, gearbox replacements, the downtime loss of power generation, and so on [1,2,3]. To increase the competitiveness of wind power compared to conventional sources, methods to further lower the relatively high OM costs of wind turbine systems are needed [1]. Condition monitoring techniques offer potential solutions by eliminating the costly machine shutdowns for inspection, avoiding catastrophic component failure during operation, and facilitating the effective scheduling of preventive maintenance [3]. The condition monitoring problem in wind turbines is a broad area of research. This problem can be dealt with by either sensor-based methods or analytical model-based methods, or a combination of those as hybrid methods. This paper describes a sensor-based method for the condition monitoring of wind turbine gearboxes [4,5,6,7,8]. The most commonly used sensor-based methods involve vibration monitoring, temperature monitoring, and oil debris monitoring [2,9,10]. However, studies have revealed that most failures of the gearbox occur in the bearings and gears, both of which generate wear debris in the oil lubrication system, so oil debris monitoring is thus a more direct measurement of gear or bearing faults, compared to secondary effects such as the vibration and the temperature, and has proven to be more suitable for the early detection of gearbox damage in wind turbines [3,11].

Typically, different wear conditions correspond to different wear debris sizes. In the normal operation of a wind turbine gearbox, the debris size is approximately between 1 μm and 10 μm [11]. When the gearbox enters abnormal conditions, debris between 10 μm and 50 μm is detected [12]. If the debris size is larger than 100 μm, the gearbox is considered to be in critical condition with a high probability of failure; this indicates that the immediate machine maintenance is needed to avoid machine failures. Thus, to detect gearbox damage early and effectively, debris sizes between 10 μm and 100 μm should be monitored. Moreover, with the application of new materials in friction pairs, such as ceramic bearings, the sensor should also have the capability to detect non-ferrous debris [13]. At present, the main online methods for oil debris monitoring are the chip detector and the inductive sensor. However, due to the restrictions of their monitoring principles, chip detectors are incapable of capturing non-ferrous debris and inductive devices are also insensitive to smaller debris and non-ferrous debris. The detection range of inductive devices is typically >100 μm ferrous and >250 μm non-ferrous debris [14,15]. To solve these problems, the oil-line electrostatic sensor (OLES) was developed.

Wood from Southampton University first proposed the electrostatic induction method at the World Tribology Conference in 1997, and developed a prototype sensor in an attempt to monitor the oil-lubricated gear gluing or adhesive wear [16,17]. The basic principle of this method is that the debris produced by the failure of components in the oil system will carry a certain amount of charge, which will be monitored by the electrostatic sensor. Experiments have shown that the OLES is sensitive to debris with sizes as small as 20 μm, and has the same capability to monitor metallic and non-metallic particulates [18].

To develop this new debris monitoring technology, a series of experiments were conducted to explain the mechanism of charge generation in the lubrication system. Through experiments, Harvey found the debris produced by adhesive wear to be positively charged and the magnitude of charge was directly related to the total volume loss; the higher the total volume loss, the larger the charge [19]. Wood presented an experimental investigation into the effect of lubricating oil quality on tribo-charging, indicating that the oil flow would also carry some charge [20]. In addition, Harvey designed a test rig and studied the effects of oil chemistry on the charging ability of various oils [21]. Morris investigated how the component material affected the charge mechanisms [22]. Also, Wang conducted research on ceramic materials and the quantitative relationship between charge level and wear volume [23].

In the field of sensor applications, many laboratory-based simulation platforms were built to verify the feasibility of this sensor. Harvey developed a gear rig test (FZG) instrumented with electrostatic sensors to simulate its practical application, in which debris was detected and confirmed by a posterior test analysis of debris captured in a magnetic filter [20]. Moreover, a bearing rig test was studied to investigate the use of electrostatic monitoring for taper roller bearing applications [24]. Both the literature and experiments have shown that in the laboratory environment, favorable factors such as a good measurement environment and an ideal simulation test platform enable electrostatic sensors to achieve good measurement results. Compared to laboratory tests, however, there are few studies on real industrial applications. The most famous application is the engine test conducted by Pratt and Whitney. Two F100 seeded fault engine tests (SFETs) were performed to explore the practical application of OLES. However, in their experiment, the sensor did not really detect the debris [25].

As the engine test showed, in real industrial environments, the complex electromagnetic environment, the high-power lubricating oil circuit, and the particularity of the structure of each test object often result in different signal characteristics compared to those observed in the laboratory environment. Therefore, to apply electrostatic sensors to wind turbine gearboxes, the effectiveness of the sensor under actual application environments must be studied in-depth. To this end, this paper focuses on the development of the new wear debris recognition method suitable for practical application environments, and the experimental research of the signal characteristics under practical application conditions is also conducted. The rest of this paper is organized as follows: the second section mainly introduces the principle of the electrostatic sensor, and proposes the debris recognition algorithm based on the two-probes structure. The third section introduces the test device and the test process. The fourth section gives a comprehensive analysis of the test results. The fifth section is the conclusions.

## 2. The Electrostatic Sensor

**Figure 1:**
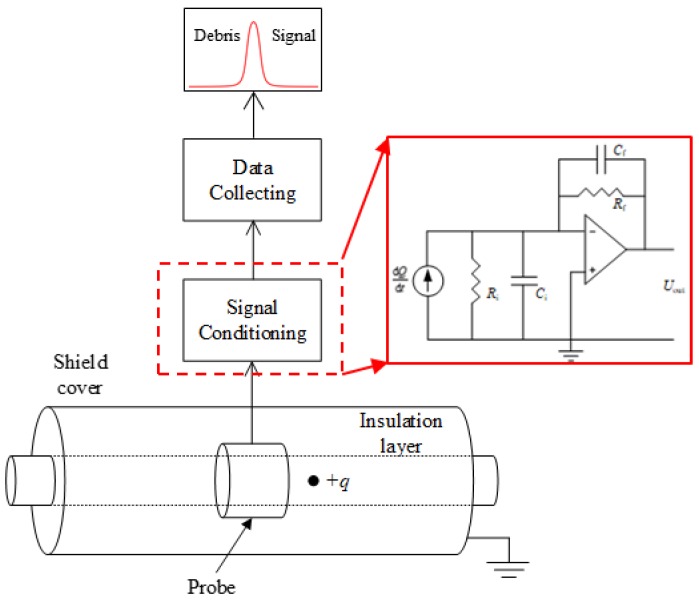
The physical structure and the data acquisition system block diagram of an oil-line electrostatic sensor (OLES).

The oil-line electrostatic sensor (OLES) is a full-flow electrostatic induction device that is installed in line with the lubricating oil system. The physical structure of the electrostatic sensor is shown in Figure 1. The sensor consists of an insulating inner tube, a probe, and a shield. The shield is grounded to shield the sensor from the interference of external electrostatic fields; the sensing probe is connected to the signal conditioning circuit, and the most basic signal conditioning circuit is a charge amplification circuit, which can convert the charge signal into a voltage signal so that it can be easily collected by the subsequent data acquisition circuit. The principle of the charge amplification circuit is also presented in Figure 1. According to the virtual ground principle, the relationship between the output voltage of the amplifier and the induced charge is:

(1) $$U_{out}=-\frac{Q}{C_{f}}$$

Here, *C_f_* is the feedback capacitance of the amplifying circuit, *U_out_* is the output voltage, and *Q* is the charge induced on the probe. When the charged particles pass through the sensor, due to the electrostatic induction principle, the probe surface will have a certain induced-charge on it, which is then amplified by the charge amplifier and converted into digital signals by the data acquisition circuit for subsequent analysis.

### 2.1. Mathematical Model of the Electrostatic Sensor

**Figure 2:**
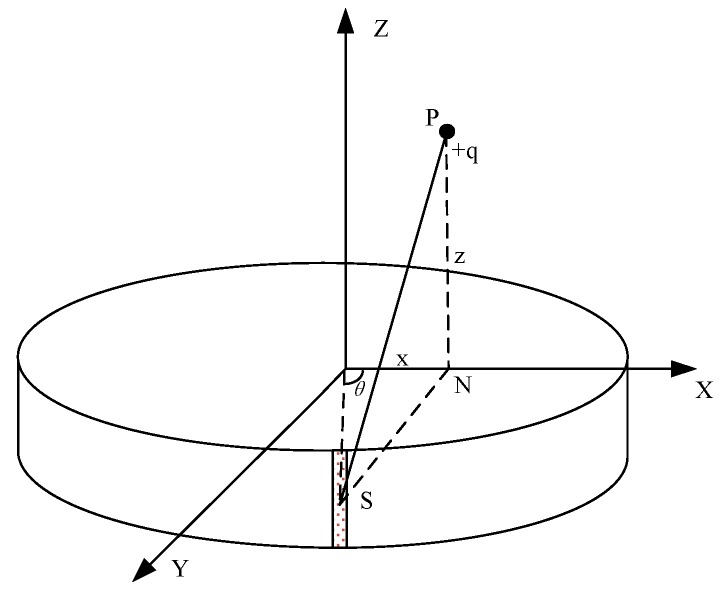
The coordinate system used in the model.

In order to develop the wear debris recognition method based on the electrostatic sensor, it is necessary to fully understand the performance of the sensor. Establishing the mathematical model of the sensor is a good way to determine its performance. According to the structure of the sensor, the mathematical model of the OLES can be simplified as a ring probe and a point charge. For the case of multiple wear debris, by the Gaussian Theorem, it can be calculated by the superposition of the single wear debris. The mathematical model of a ring probe and a point charge is described in detail in Reference [26], and the output signal of the sensor is defined as Equation (2), which has been proven both theoretically and experimentally. This paper mainly focuses on the research of the measurement of debris, so the mathematical model in [26] is used directly. For the sake of completeness, the coordinate system in the model is presented in Figure 2. The coordinate system takes the axis direction of sensor as the zenith direction, the center of probe as the origin, and the plane orthogonal to the zenith and containing the origin as the XOY plane. The mathematical model is as follows:

(2) $$Q=-\frac{Dq}{4\pi }\int_{0}^{\pi }\frac{0.5D-x\cos\theta }{F^{2}(x,\theta )}\times (\frac{z+0.5W}{{\left[{\left(z+0.5W\right)}^{2}+F^{2}(x,\theta )\right]}^{1/2}}-\frac{z-0.5W}{{\left[{\left(z-0.5W\right)}^{2}+F^{2}(x,\theta )\right]}^{1/2}})d\theta$$

where *q* is the charge of debris; *D* and *W* are the diameter and axial length of the electrode respectively; *θ* is the azimuth angle of the micro-element sensing region; *z* is the coordinate of the debris in the Z direction; *x* is the coordinate in the *X* direction; and F(x,θ) indicates the distance between the projection N on the XOY plane of the debris and the micro-element *S* of the probe, which is a function of *x* and *θ*:

(3) $$F(x,\theta )=\left|SN\right|={\left[{\left(0.5D\right)}^{2}+x^{2}-Dx\cos\theta \right]}^{1/2}$$

**Figure 3:**
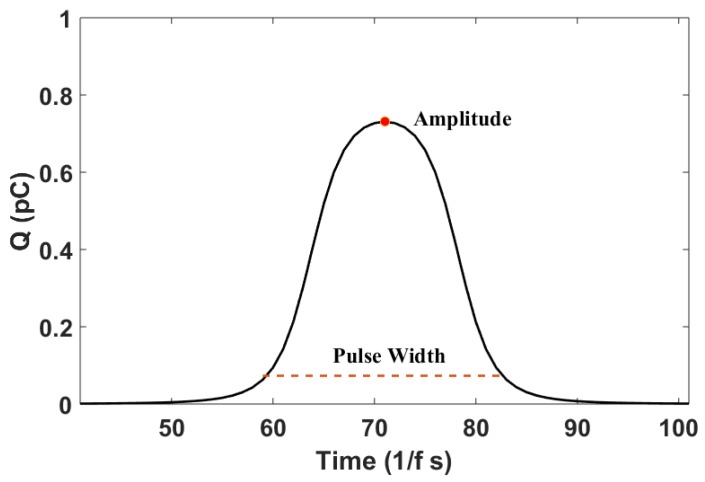
The simulated waveform.

On the basis of the sensor’s mathematical model, the waveform of the signal can be determined by numerical simulation, as shown in Figure 3. From the simulated waveform, it can be seen that the debris characteristic signal is similar to a bell-shaped pulse, and the waveform has a certain amplitude *U_peak_* and width *τ_width_*. In this paper, for the convenience of identification, the pulse width *τ_width_* is defined as the time between the points with a value equal to 0.1 times *U_peak_*. Thus, the amplitude *U_peak_* and the pulse width *τ_width_* can be extracted as features to indicate the debris. Meanwhile, according to the mathematical model, the amplitude of the characteristic waveform is related to the quantity of charge *q* carried on the debris, and the greater the charge, the larger the amplitude. The pulse width of the characteristic waveform has a relationship with the speed of the debris passing through the sensing area of the sensor; the greater the speed, the smaller the pulse width.

### 2.2. Debris Recognition Method using Electrostatic Signals

**Figure 4:**
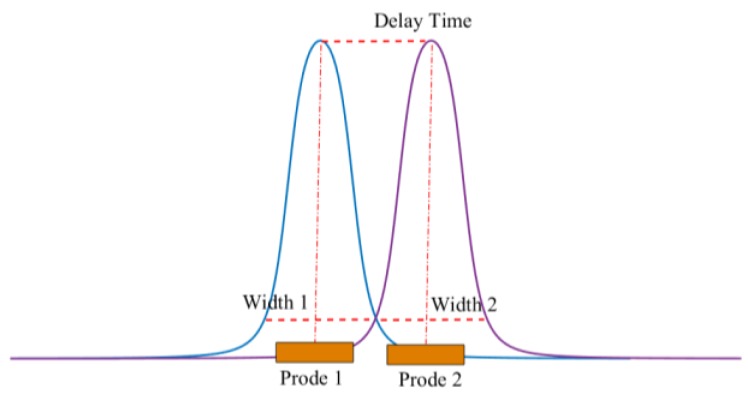
The two probes structure.

Through the above analysis, the pulse amplitude *U_peak_* and pulse width *τ_width_* of the debris characteristic signal can be used to identify the debris. However, in the actual industrial environment, serious interference problems may result in poor recognition accuracy of the characteristic pulse. The electrostatic signal belongs to weak signals, and the acquisition of weak signals is easily interfered by the noise. Firstly, some electromagnetic interferences in the noise can also generate waveforms similar to the debris characteristic pulse, thus affecting the recognition accuracy. Secondly, strong noise can cause the debris characteristic pulse to be submerged in the noise signal and become difficult to identify. Some de-noising method can improve the signal to noise ratio (SNR), but only to a small extent. For the traditional electrostatic sensor with one-probe as shown in Figure 1, in order to improve the recognition accuracy, it is necessary to set a high pulse recognition threshold to ensure that the signal is caused by debris rather than noise, which limits the detection ability of debris with low amplitude. To solve these problems, this paper proposes a method based on a sensor with two probes. As shown in Figure 4, since the two probes are installed successively in the flow direction, the debris will sequentially induce the characteristic pulse on the two probes. The delay time between the pulses is related to the moving speed of the debris and the distance between the two probes, which can be expressed as:

(4) $$\tau_{delay}=\frac{D_{probe}}{v_{debris}}$$

**Figure 5:**
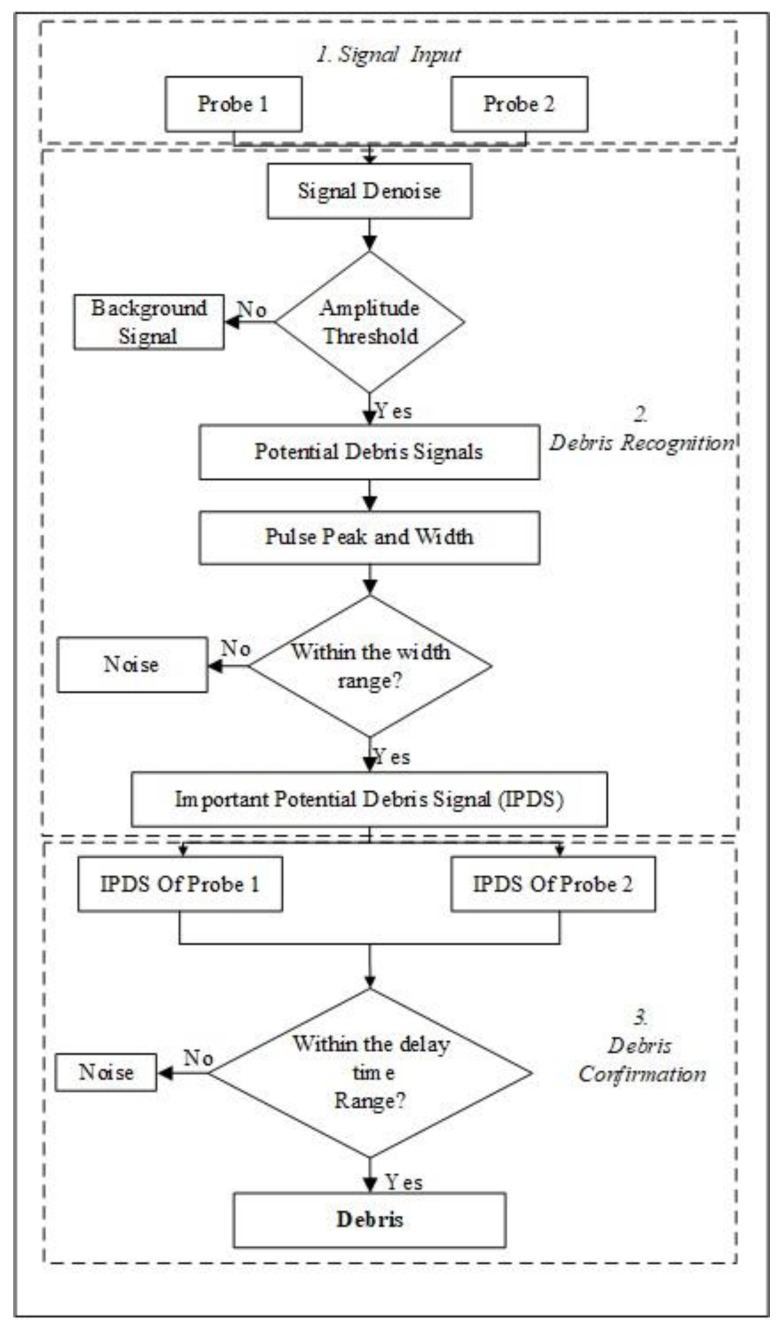
Debris identification method.

**Figure 6:**
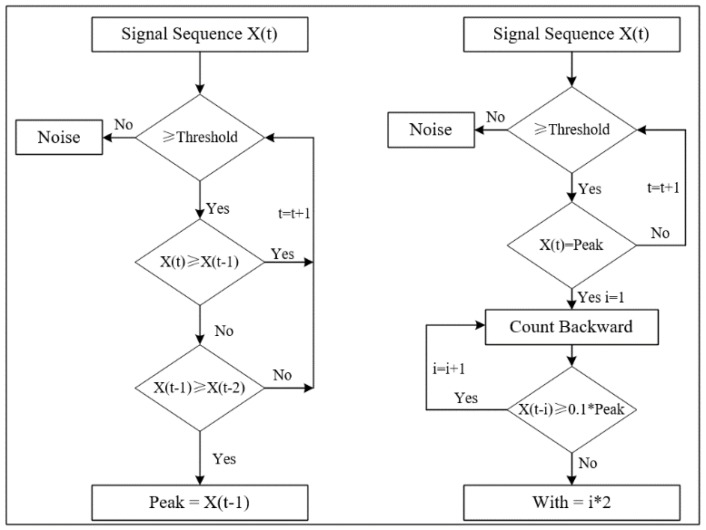
Peak value and pulse width identification methods.

Here, *τ_delay_* means the delay time, *D_probe_* is the distance between the two probes, and *ν_debris_* is the velocity of the debris. Using the features *U_peak_*, *τ_width_* and *τ_delay_*, the detailed recognition algorithm is shown in Figure 5 and Figure 6 and a detailed description follows.

Step 1: Input the signals of the two probes.
Step 2: Noise reduction. As the electrostatic signals belong to weak signals, strong noise can cause the debris characteristic pulse signal to be submerged in the noise and become difficult to identify. In order to improve the recognition accuracy, it is necessary to perform noise reduction first. In this paper, the main noise in the signal is the power frequency interference of 50 Hz. Therefore, a Finite Impulse Response (FIR) digital notch filter is used to eliminate the power frequency interference, the stop-band of which is set to 45–55 Hz.
Step 3: Identify the Potential Debris Signal. To distinguish between the debris signal and the random noise signal, an amplitude threshold is set according to the background signal magnitude level. The signal above threshold is considered to be the Potential Debris Signal and the signal below threshold is considered to be the noise.
Step 4: Identify the Peak and Pulse width of the Potential Debris Signal. The detailed identification method is presented in Figure 6. For the pulse peak, the identification method is obvious. After finding the peak value, count the signal sequence backward until it reaches 0.1 times the peak value. The resulting count value multiplied by twice the sampling interval is the width of the debris characteristic pulse.
Step 5: Identify the Important Potential Debris Signal (IPDS). According to the pulse width, determine whether the signal is caused by debris or interference. For interference, its pulse width is usually very short. For the debris signal, the pulse width has a certain range corresponding to the flow rate, which can be determined by the analysis of the model and experiments. A pulse width threshold is set to distinguish the interference from the potential debris signal. The signal within the width range is set as the IPDS, and the signal below the lower limit of the width range is considered to be the noise.
Step 6: Conform the debris signals with the two probes. As the debris passes through the first probe and the second probe, there is a delay time between the occurrence time of the debris signals in the two probes. Whereas, if the IPDS is caused by the interference, the IPDS will occur in the two probes at the same time. Therefore, by verifying the delay time between the IPDSs in the two probes, the debris can be finally confirmed.

In the case when multiple pieces of debris pass through the sensor at the same time, the superposition of the signal will widen the pulse width (above the upper limit of the range determined by the analysis of its model and experiments), thus causing it to be isolated to provide more in-depth identification information. Here, we use a formula to correct large pulse width signals:

(5) $$number\;of\;debris=\left\lceil \frac{the\;measured\;width}{\max(the\;range\;of\;the\;width)}\right\rceil$$

Compared to the sensor with one probe, the new method can distinguish the interference and the debris signals better. Moreover, for the sensor with two probes, because the signals from the two probes can verify with each other using the delay time, the pulse amplitude recognition threshold can be set lower, which is beneficial to improve the recognition rate of the debris.

## 3. Experimental Setup and Test Procedure

### 3.1. Experimental Setup

#### 3.1.1. Gearbox Bench Test Device

**Figure 7:**
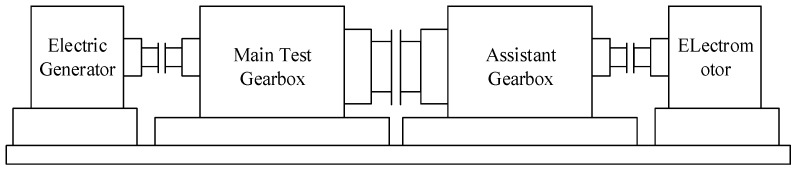
Schematic of the gearbox test rig.

This experiment was performed in the wind turbine gearbox bench test. The test device is shown in Figure 7, and consists of two actual 2.1 MW wind turbine gearboxes, two serial drive motors, and two serial loading motors. The test system controls the output speed and torque of the drive unit through the frequency converter to realize the simulation of different working conditions and to complete the test of the gearbox. The test gearbox is a typical wind turbine gearbox with three stages of gearing—a high speed stage, an intermediate stage, and a planetary stage.

#### 3.1.2. Electrostatic Monitoring System

**Figure 8:**
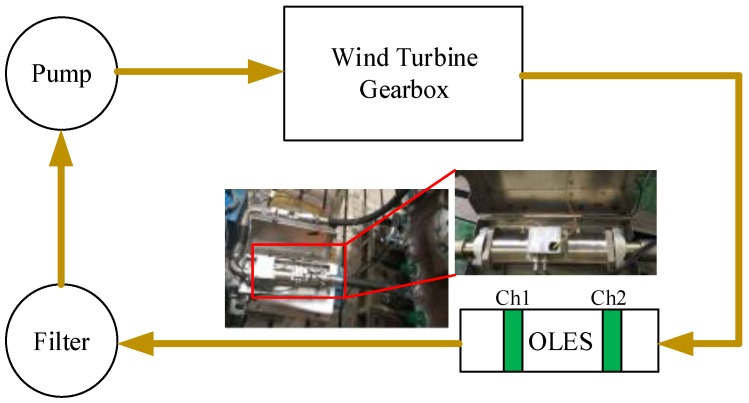
OLES installation diagram.

An OLES was mounted in the oil recirculation system of the main test gearbox, just downstream of the test gearbox, as shown in Figure 8. The sensor comprises two full ring probes, spaced 100 mm apart and flush with the pipe outer wall. To minimize the leakage of the charge on the debris and other influences, the OLES was installed in a shielding box, as close as possible to the gearbox. A filter after the OLES holds all particles larger than 50 μm, preventing them from re-entering the oil circuit. The sensitivity of the OLES is 0.6.

Two signal conditioner units were used to convert the electrostatic charge induced on the sensor into a voltage signal. Both units have a frequency response from 1 Hz to 5 KHz. Channel gains were set to 100 mV/pC. The data acquisition system was realized by the NI c-DAQ 9191 network chassis and the NI-9234 data acquisition card (National Instruments, Austin, TX, USA) assisted by special software developed in LabVIEW. The sampling frequency of the system was set to 2 KHz. All data were stored in the computer for subsequent analysis. Signal processing was carried out using MATLAB.

### 3.2. Test Procedure

The test is the Highly Accelerated Life Testing (HALT) to evaluate the effective life of the gearbox. The gearbox was loaded nine times in total, and the speed was kept constant under each load. Only the applied load of the gearbox was increased and the test time was adjusted. The specific load status is shown in Table 1.

## 4. Results and Analysis

### 4.1. The Raw Signal

#### 4.1.1. The Debris Characteristic Signal

**Figure 9:**
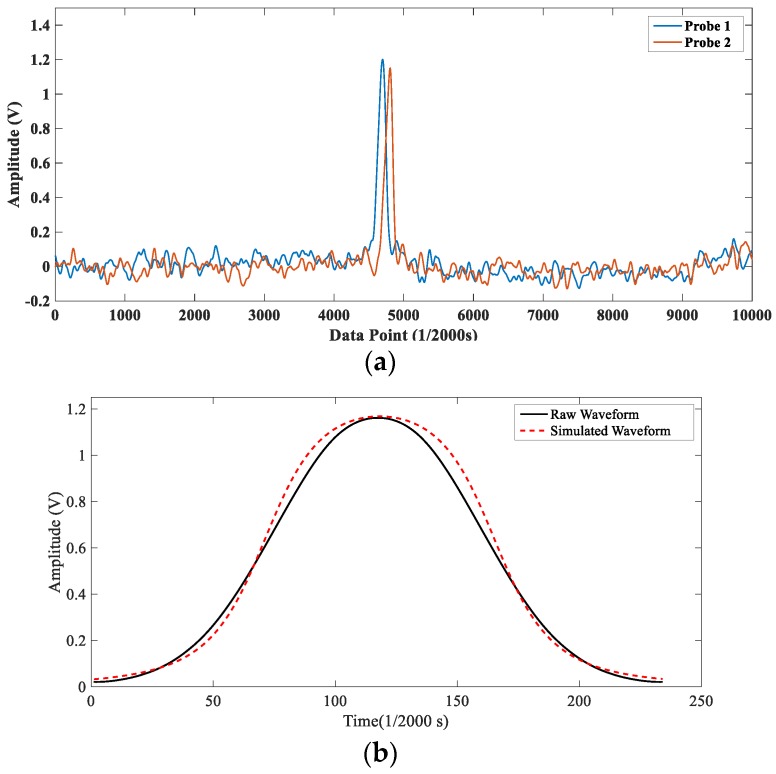
The raw signal with the debris characteristic pulse. (**a**) The raw data with the debris characteristic pulse; (**b**) The debris characteristic pulse.

Figure 9a shows a raw signal containing a characteristic pulse of the wear debris, and Figure 9b depicts its amplified waveform. In Figure 9b, it is observed that the actual waveform is very similar to the simulated waveform in Section 2 and the correlation coefficient calculated by the two waveforms is 0.9973, which proves the correctness of the mathematic model qualitatively. Moreover, in Figure 9a the delay time between the two pulses is obvious, which indicates that the debris recognition algorithm proposed in Section 2 is also feasible.

#### 4.1.2. The Background Signal

**Figure 10:**
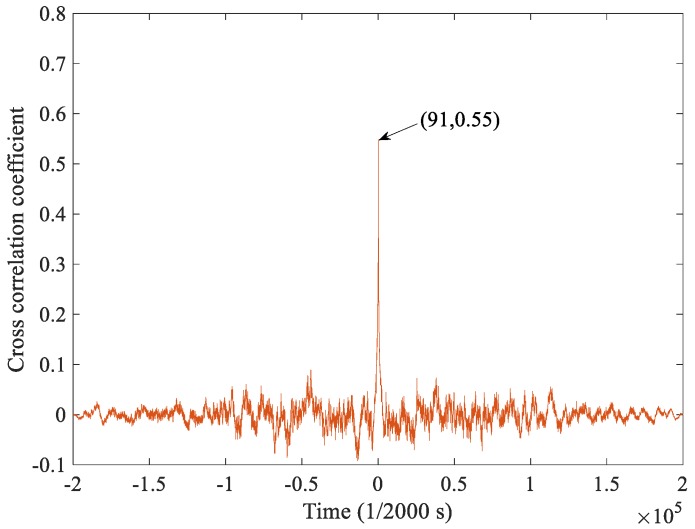
The result of the cross correlation coefficient between the two probes.

It was noticed that in the raw signal, even if there is no debris generated, the background signal has a relatively large amplitude. This phenomenon may be related to the charging of the oil flow in addition to the interference. To prove this assumption, the correlation between the two channels was calculated. As shown in Figure 10, the result of the cross-correlation coefficient shows that there is a significant correlation between the two signals with a delay time of 91/2000 s. It is known that the flow rate of the lubricating oil is about 150 L/min and the distance between the two probes is 0.1 m, so the flow velocity of the lubricating oil can be converted to be about 2.2 m/s in the pipe with a diameter of 38 mm, and thus the time it takes for the oil to flow through the front and rear probes is just 0.0455 s. The same results indicate that the background signal is related to the oil flow and may be related to the oil-flow charge, as mentioned in the introduction.

**Figure 11:**
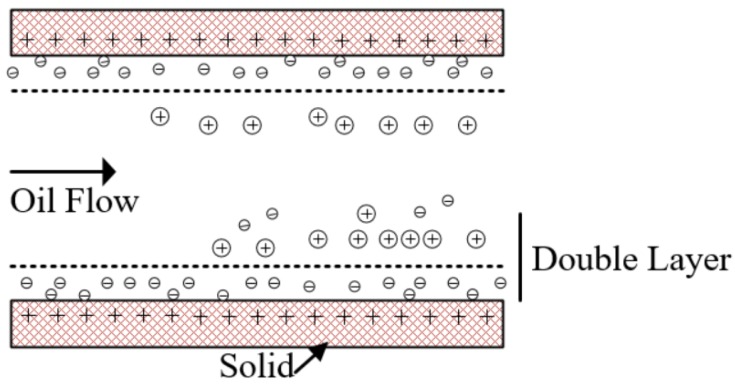
The Double Layer in the interface of the tube and oil.

For the oil-flow charge, studies have shown that, in the lubricating oil system the oil’s relative motion over a solid surface will lead the oil to carry some charge, which is a well-known electrochemical concept named tribo-charging [27,28]. Tribo-charging can be used to explain in part why oil flows are charged. There is a double layer existing in the interface of the oil tube and the low conductivity oil, as shown in Figure 11. The oil layer closest to the solid surface consists of either all positive or all negative charges. When an oil with low conductivity flows over the solid surface, the relative motion will lead a portion of the double layer being stripped and entrained into the oil flow, thus generating free electrostatic charge in the oil flow. In the stable operation condition of gearbox in this test, the charge carried by the oil flow corresponded to 1.6 pC. Unlike the noise, the background signal caused by oil flow cannot be reduced, so the amplitude of the debris characteristic pulse must be higher than the background signal to ensure that the debris is effectively recognized. However, the experiment found that the background signal is not fixed. When the amplitude of the background signal exceeds the amplitudes of most debris pulses, the sensor will lose efficacy. Therefore, it is necessary to study the factors affecting the background signals.

#### 4.1.3. Background Signals in Different Conditions

**Figure 12:**
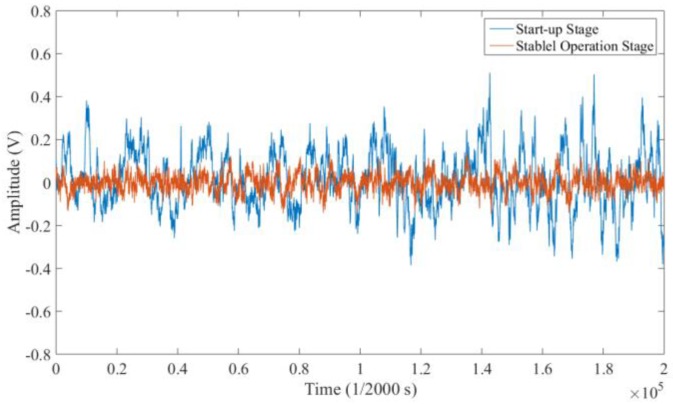
The raw signal in the start-up stage and the stable operation stage.

During the test, it was observed from the raw signals that the magnitude of the background signal varied in different operating conditions. At the beginning of the operation, the fluctuation range reached ±0.4 V, as shown in Figure 12. However, after the gearbox entered into the stable operation condition, the background signal kept stable with an amplitude within ±0.1 V. This phenomenon may be caused by the different conditions of the oil and the gearbox, including the speed of the oil pump (low or high status), gearbox rotation speed, gearbox torque, and oil temperature. The following is the detailed analysis of the signal characteristics under each working condition.

##### The Status of the Lubricating Oil Pump

**Figure 13:**
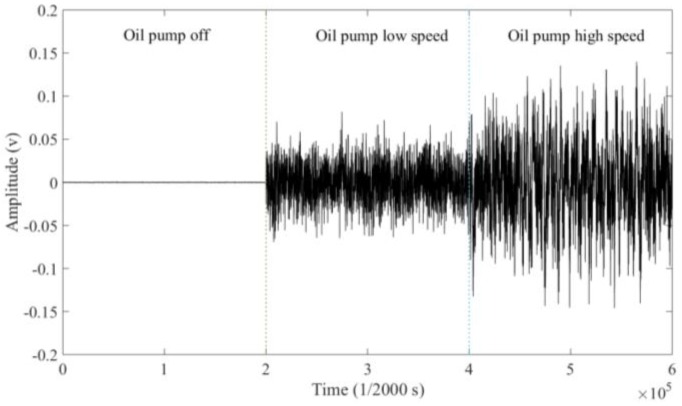
Background signals in different statuses of the lubricating oil pump.

The oil pump controls the oil flow rate, which has three statuses: off, low speed and high speed. As can be seen from Figure 13, under different working conditions of the lubricating oil pump, the signals are significantly different. When the high-speed pump is on, the oil flow rate is high and the corresponding background signal is large; while in the low-speed status, the oil flow rate is low and the corresponding background signal is small. The experimental results reveal that there is a positive correlation between the flow rate and the amplitude of the background signal.

##### Oil Temperature

**Figure 14:**
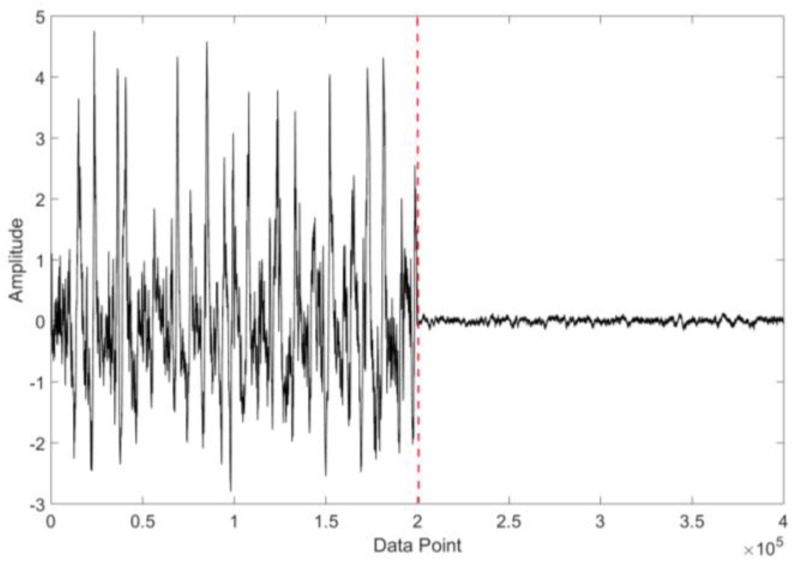
The signals in the low oil temperature and the high oil temperature.

Figure 14 shows the raw background signals of the electrostatic sensor at a low oil temperature (17 °C) and a high oil temperature (42 °C). As shown, in the low oil temperature state (17 °C), the amplitude range of the background signal of the sensor is very large, reaching ±3 V; but in the high oil temperature status (42 °C), the amplitude range is only ±0.05 V. The relationship between the background signal amplitude and the oil temperature is further analyzed in detail as follows.

At the beginning of the 100% load test, the torque is stabilized at 100% load, but the oil temperature is still low due to the cold environment temperature, and the oil temperature gradually rises as the gearbox continues to operate. The data at this stage excludes other influences of the working conditions and can be used to analyze the influence of temperature on the electrostatic signal in detail. In order to eliminate the influence of the polarity of the charge, the Root Mean Square (RMS) value of the electrostatic signal was calculated to represent the magnitude of the background signal. The specific test conditions are listed in Table 2.

**Figure 15:**
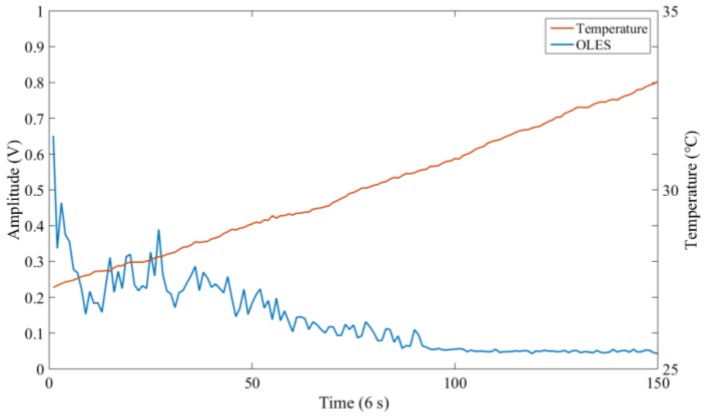
The relation between the electrostatic signal and temperature.

Figure 15 depicts the relationship between the electrostatic signal and temperature. It can be clearly seen that, as the temperature rises, the electrostatic signal slowly decreases. Although there is a fluctuation at the beginning, the overall trend is downward. When the temperature reaches a certain value (30.5 °C in this test), the electrostatic signal stops falling and the RMS value remains around 0.048. As known, the oil viscosity decreases with temperature. Considering the mechanism of the oil flow charging, it is highly possible that the oil-flow charge is related to the viscosity of the oil. When the oil temperature was low, high viscosity of the oil would result in a large tribo-charge. Meanwhile, when the viscosity reached a certain value, the effect on the oil flow charging would be no longer obvious.

##### Effect of the Gearbox Torque on the Background Signal

Next, we further analyzed the effect of the gearbox torque on the electrostatic signal. The signals of four stages 20%, 40%, 60% and 80% load were selected. In these selected conditions, the speed and temperature are kept stable and the oil pump is at a high speed status. The specific working conditions are shown in Table 3.

**Figure 16:**
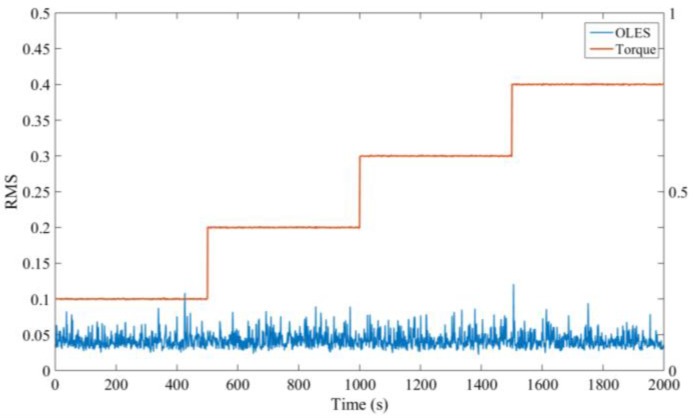
The variation of the electrostatic signal with torque.

The RMS value of the signal monitored by the sensor at each stage is shown in Figure 16. It shows that the electrostatic signal does not change with the loads, and the RMS value of the electrostatic signal remains stable at around 0.05.

##### Effects of the Gearbox Rotation Speed on the Electrostatic Signals

**Figure 17:**
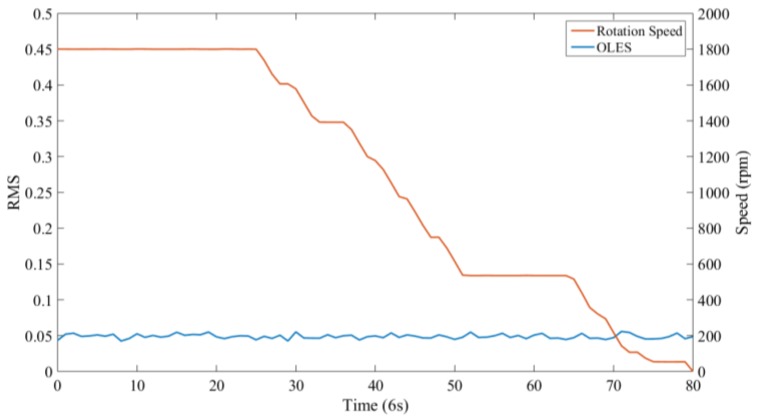
The relationship between the electrostatic signal and the gearbox rotation speed.

The signal of a de-loading stage was selected for the analysis. At this period, the load and oil temperature both remain unchanged. The specific working conditions are shown in Table 4. The signal monitored by the sensor at each speed is shown in Figure 17, which indicates that the magnitude of the background signal does not substantially change with the rotational speed of the gearbox.

Therefore, it can be concluded from this experiment that the electrostatic signal contains a certain amplitude of the background signal due to the charging of the oil flow, which is related to the oil temperature and the flow rate, but has no relationship with the operating conditions of the gearbox. Since the oil pump basically maintains stable operation (high speed or low speed), only oil temperature has a relatively large effect on the signal. In the start-up phase of the gearbox, as the oil temperature is low, the background signal of the sensor fluctuates greatly, exceeding the amplitudes of most debris characteristic pulses, which will cause the sensor to lose efficacy at this stage. However, with the operation of the gearbox, the oil temperature rises rapidly and then remains substantially stable under the action of the cooling system of the gearbox. Under the high and stable oil temperature condition, the background signal of the sensor will restore stability soon and the sensor function will also recover. Therefore, the electrostatic sensor will be effective under almost all operating conditions except the short start-up phase, which indicates that the sensor has a strong applicability in the wind turbine gearbox.

### 4.2. Wear Debris Analysis

**Figure 18:**
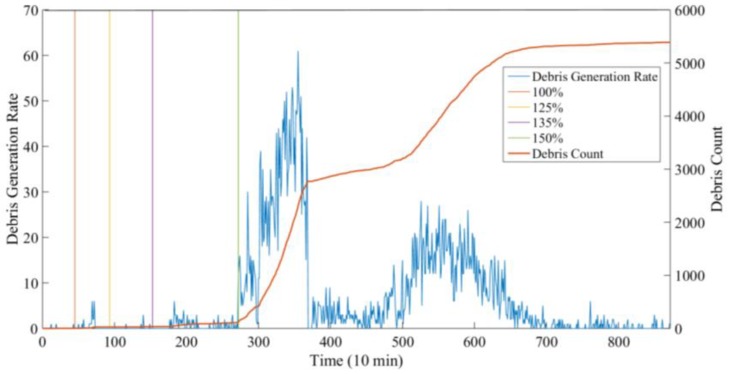
Debris recognition results.

To minimize the disturbance caused by the unstable operation condition of the gearbox during startup phases, only the signals from the stable operation periods were used for the debris identification. Using the identification method proposed in Section 2, the result of the debris count is shown in Figure 18. In Figure 18, the abscissa is the time series, and each point represents 10 min; the ordinate ‘Debris Generation Rate’ indicates the amount of debris generated in each time unit (10 min), while the ordinate ‘Debris Count’ means the accumulated amount of the debris until the time point.

As the recognition results show, the debris count in the 20~100% load phase remained almost unchanged, indicating that the gearbox condition was possibly health at this time and the debris were mainly released from benign wear. From the 125~150% load stage, the amount of particles began to increase but still remained at a relatively low level. By the beginning of the 170% load stage, the debris count rose rapidly and remained at a very high level for nearly 17 h. This phase was most likely to be related to the early stages of damage. At around 400 × 10 min, a routine inspection found slight pitting on the gear tooth of the intermediate stage. Subsequently, at 480~600 × 10 min, the amount of the debris increased again. During this period, it is highly probable that the damage was further expanded. After the test, the inspection revealed that pitting area had expanded as monitored by the electrostatic sensor, which proved the effectiveness of the sensor.

### 4.3. Wear Debris Size Analysis

Section 4.2 gives a recognition result for the presence of the wear debris, but the size information of the debris was not yet obtained. It is known that the morphological information of the debris can provide more fault information of the gearbox. This section attempts to analyze the size information of the debris. Studies have shown that the charge of the wear particles is positively correlated with its sizes; that is, the larger the size of the wear particles, the more the particles are charged. An empirical equation is given in the literature [29], which is:

(6) $$Q_{p}=\alpha \cdot d_{p}{}^{\gamma }$$

where: *α* is a constant associated with the material of the debris and the medium the debris in; is the equivalent diameter of the particle; and *γ* is the constant determined by the test, the value of which usually ranges from 1.2 to 1.6. Taking debris with an equivalent diameter of 100 μm as an example, its charge would be about 10 pC.

**Figure 19:**
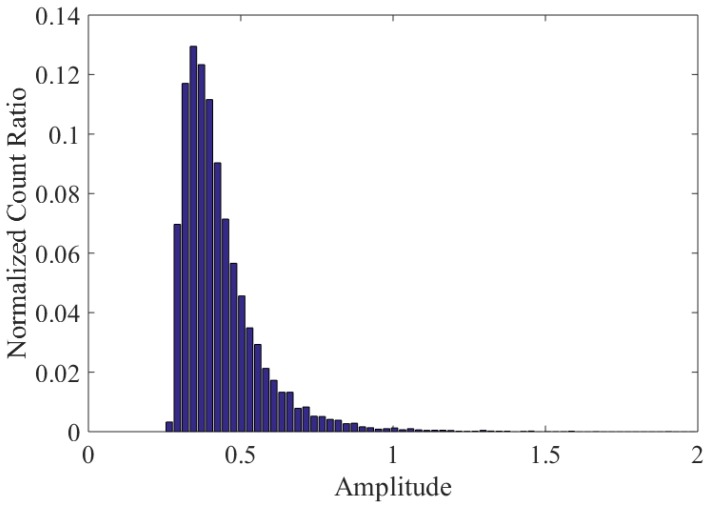
The distribution of the amplitudes.

To reveal the size information from the amplitudes in this test, the distribution of the amplitudes of all debris characteristic signals was analyzed, as shown in Figure 19. It was observed that the amplitude is mainly concentrated in the low amplitude range (0.3 V, 0.4 V). Besides, the number of debris and the amplitude range are negatively correlated; the larger the amplitude range, the fewer the number of wear debris. This is consistent with the gearbox wear debris size distribution rule presented in [9,11].

**Figure 20:**
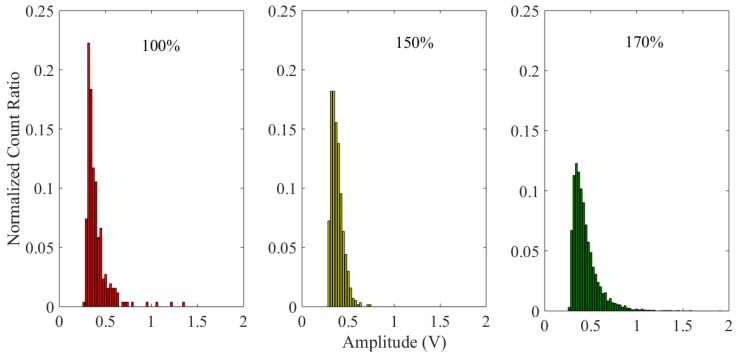
The normalized count ratio in different operation phases.

In addition, the distributions of characteristic pulse amplitudes at different life stages also differed among each other, as shown in Figure 20. At the beginning of the test, the amplitude of the characteristic pulses mainly concentrates on the low amplitude (0.32 V 0.33 V). As the test progresses, the ratio of the low characteristic pulse amplitude decreases, but the proportion of high amplitude increases. At the end of the experiment, these trends tend to be more obvious. Furthermore, the maximum ratio value was significantly shifted to a higher interval (0.34 V 0.35 V). This is also consistent with the change rules of the wear debris size and indicates the damage is increasing.

**Figure 21:**
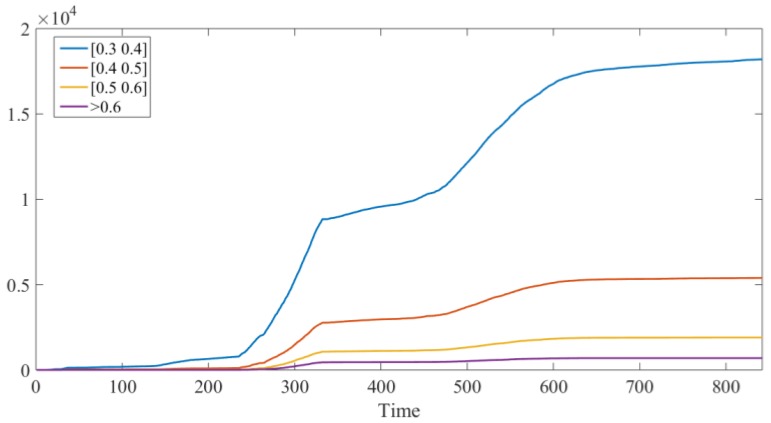
Debris counts in different amplitude intervals.

An additional statistical analysis of the debris count trend in each interval with time is shown in Figure 21. This figure shows that the trends of the debris count in each interval are basically the same, but in the low amplitude interval, the wear debris growth is more obvious. The moment when the wear debris abnormally increases is very likely to correspond to the moment when a fault occurs. Therefore, the statistics of the low debris characteristic pulse amplitudes are significant to improve the sensitivity of sensors for anomaly monitoring.

### 4.4. Charge Leakage Analysis

**Figure 22:**
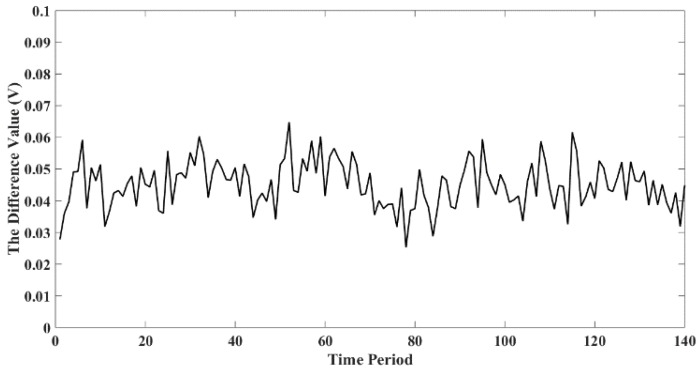
The amplitude difference between the first probe and the second probe.

The experiment also found that the amplitude of the signal detected by the second probe of the sensor is usually smaller than that of the first probe. This means that charged wear debris is most likely to leak in the oil. Although the oil is an electrostatic non-conductor, the additive ions and impurities contained in the oil may lead to leakage of the charge carried by the wear debris. Figure 22 shows the difference between the amplitudes of the first probe and the second probe over a period of time, with fluctuating values around 0.043 V. Based on the results obtained, it would be ideal to install the sensor as close as possible to the gearbox in the practical application.

## 5. Conclusions

In this study, a novel debris recognition method based on an electrostatic sensor with two probes was developed and the characteristics of the sensor under practical application environments were researched. With the wind turbine gearbox bench test, the feasibility of the proposed debris recognition method and the application potential of the electrostatic sensor for wind turbine gearboxes were evaluated. The main conclusions are summarized as follows:

(1) With the proposed debris recognition method, the electrostatic sensor can capture the debris characteristic pulse effectively in the practical application. The measured result of the OLES indicated the anomalous condition of the gearbox successfully, as proven by the subsequent inspection of the gearbox.
(2) In the wind turbine gearbox bench test, the electrostatic signal contains the background signal with certain magnitudes due to the charging of the oil flow, which is related to the oil temperature and the oil flow rate, but has no relationship with the operating conditions of the gearbox. As the temperature and the flow rate of the oil remain stable after the startup phase, the electrostatic sensor is effective in all operation phases of the wind turbine except the short start-up phase, indicating that the electrostatic sensor has a strong application applicability in the wind turbine gearbox.
(3) During the whole test, the amplitude of the recognized debris pulse signal mainly concentrates in the low amplitude interval. In addition, the amplitude concentration gradually increases with machine operation time, which is consistent with the wear debris size growth rule in the gearbox. This result proves that there is a positive correlation between the wear debris size and the amplitude of the debris characteristic pulse, providing a direction for further research on the acquisition of wear debris size information by electrostatic signals.

As this paper mainly focused on the identification of the amount of debris in the wind turbine gearbox, the morphological information of the debris was not considered in-depth. In the future, the relationship between the electrostatic signals and morphological information of the debris will be further studied, such as debris sizes and materials, with the aim of improving the effectiveness of OLES for fault diagnosis.

## Figures and Tables

**Table 1:** Load stage condition.

No.	Torque (%)	Speed (rpm)	Duration (h)
1	20	1800	1
2	40	1800	1
3	60	1800	1
4	80	1800	1
5	100	1800	1.5
6	125	1800	10
7	135	1800	10
8	150	1800	20
9	170	1800	100

**Table 2:** Test condition.

Speed (rpm)	Torque	Initial Temperature of Oil (°C)	End Temperature of Oil (°C)
1800	100%	27.23	33.03

**Table 3:** Test condition.

No.	Torque	Speed (rpm)	State of the Oil Pump	Oil TEMPERATURE (°C)
1	20%	1800	High	44.32
2	40%	1800	High	44.21
3	60%	1800	High	44.97
4	80%	1800	High	45.51

**Table 4:** Test condition.

State of the Oil Pump	Torque (Nm)	Oil Temperature (°C)
High	2400	33.46

## Nomenclature

OLES	Oil-line electrostatic sensor
FZG	A gear rig test
SFETs	Seeded fault engine tests
FIR	Finite Impulse Response
IPDS	Important potential debris signal
*U_out_*	The output voltage of the charge amplification circuit
*Q*	The charge induced on the probe by the debris
*C_f_*	The feedback capacitance of the amplifying circuit
*q*	The charge of the debris
*D*	The diameter of the probe
*W*	The axial length of the probe
*θ*	The azimuth angle of the micro-element sensing region S in the coordinate system of the model
*z*	The coordinate of the debris in the Z direction
*x*	The coordinate in the X direction
*F*(*x*, *θ*)	The distance between the projection N on the XOY plane of the debris and the micro-element S
U_peak_	The peak value of the debris characteristic pulse
$\tau_{width}$	The pulse width of the debris characteristic pulse
$\tau_{delay}$	The delay time between the two pulses on the two probes induced by the debris
*D_probe_*	The distance between the two probes
$v_{debris}$	The velocity of the debris
*Q_p_*	The charge of the particle
*α*	A constant associated with the material of the debris and the medium the debris in
*d_p_*	The equivalent diameter of the particle
*γ*	The constant determined by the test

## References

[1] Nie M., Wang L. (2013). Review of Condition Monitoring and Fault Diagnosis Technologies for Wind Turbine Gearbox. Procedia CIRP.

[2] Lu B., Li Y.Y., Wu X., Yang Z.Z. A review of recent advances in wind turbine condition monitoring and fault diagnosis. Proceedings of the 2009 IEEE Power Electronics and Machines in Wind Applications.

[3] Dupuis R. Application of Oil Debris Monitoring for Wind Turbine Gearbox Prognostics and Health Management. Proceedings of the Annual Conference of the Prognostics and Health Management Society.

[4] Tabatabaeipour S.M., Odgaard P.F., Bak T., Stoustrup J. (2012). Fault Detection of Wind Turbines with Uncertain Parameters: A Set-Membership Approach Energies. Sensors.

[5] Badihi H., Zhang Y.M., Hong H. (2014). Fuzzy Gain-Scheduled Active Fault-Tolerant Control of a Wind Turbine. J. Franklin Inst..

[6] Sanchez H., Escobet T., Puig V., Odgaard P.F. (2015). Fault Diagnosis of an Advanced Wind Turbine Benchmark Using Interval-Based ARRs and Observers. IEEE Trans. Ind. Electron..

[7] Casau P., Rosa P., Tabatabaeipour S.M., Silvestre C., Stoustrup J. (2015). A Set-Valued Approach to FDI and FTC of Wind Turbines. IEEE Trans. Control Syst. Technol..

[8] Badihi H., Zhang Y.M., Hong H. (2015). Wind Turbine Fault Diagnosis and Fault-Tolerant Torque Load Control against Actuator Faults. IEEE Trans. Control Syst. Technol..

[9] Loutas T.H., Roulias D., Pauly E. (2011). The combined use of vibration, acoustic emission and oil debris on-line monitoring towards a more effective condition monitoring of rotating machinery. Mech. Syst. Sig. Process..

[10] Ebersbach S., Peng Z., Kessissoglou N.J. (2006). The investigation of the condition and faults of a spur gearbox using vibration and wear debris analysis techniques. Wear.

[11] Zhu X., Zhong C., Zhe J. (2017). Lubricating oil conditioning sensors for online machine health monitoring—A review. Tribol. Int..

[12] Edmonds J., Resner M.S., Shkarlet K. Detection of precursor wear debris in lubrication systems. Proceedings of the 2000 IEEE Aerospace Conference.

[13] Zhan H., Song Y., Zhao H., Gu J., Yang H., Li S. (2014). Study of the sensor for on-line lubricating oil debris monitoring. Sens. Transducers.

[14] Li C., Peng J., Liang M. (2014). Enhancement of the wear particle monitoring capability of oil debris sensors using a maximal overlap discrete wavelet transform with optimal decomposition depth. Sensors.

[15] Li C., Liang M. (2011). Separation of the vibration-induced signal of oil debris for vibration monitoring. Smart Mater. Struct..

[16] Tasbaz O.D., Wood R.J.K., Browne M., Powrie H.E.G., Denuault G. (1999). Electrostatic monitoring of oil lubricated sliding point contacts for early detection of scuffing. Wear.

[17] Powrie H.E.G., Fisher C.E. Engine health monitoring: Towards total prognostics. Proceedings of the 1999 IEEE Aerospace Conference.

[18] Powrie H.E.G., Fisher C.E., Tasbaz O.D., Wood R.J.K. Performance of an Electrostatic Oil Monitoring system during an FZG gear scuffing test. Proceedings of the International Conference on Condition Monitoring.

[19] Harvey T.J., Morris S., Wang L., Wood R.J.K., Powrie H.E.G. (2007). Real-time monitoring of wear debris using electrostatic sensing techniques. J. Eng. Tribol..

[20] Harvey T.J., Wood R.J.K., Denuault G., Powrie H.E.G. (2002). Effect of oil quality on electrostatic charge generation and transport. J. Electrostat..

[21] Harvey T.J., Wood R.J.K., Powrie H.E.G., Warrens C. (2004). Charging Ability of Pure Hydrocarbons and Lubricating Oils. Tribol. Trans..

[22] Akio K., Shigenori M. (2002). Use of Electrostatic Charge Monitoring for Early Detection of Adhesive Wear in Oil Lubricated Contacts. J. Tribol..

[23] Wang L., Wood R.J.K., Care I., Powire H.E.G. (2004). Electrostatic wear sensing of ceramic-steel lubricated contacts. Tribol. Interface Eng. Ser..

[24] Harvey T.J., Wood R.J.K., Powrie H.E.G. (2007). Electrostatic wear monitoring of rolling element bearings. Wear.

[25] Powrie H. Use of electrostatic technology for aero engine oil system monitoring. Proceedings of the 2000 IEEE Aerospace Conference.

[26] Yan Y., Byrne B., Woodhead S., Coulthard J. (1995). Velocity measurement of pneumatically conveyed solids using electrodynamic sensors. Meas. Sci. Technol..

[27] Harvey T.J., Wood R.J.K., Denuault G., Powire H.E.G. (2002). Investigation of electrostatic charging mechanisms in oil lubricated tribo-contacts. Tribol. Int..

[28] Carnie S.L., Torrie G.M. (2007). The Statistical Mechanics of the Electrical Double Layer.

[29] Rossner M., Singer H. Measurement of micrometer particles by means of induced charges. Proceedings of the Conference Record of the IEEE Industry Applications Society Annual Meeting.

